# Feasibility and Safety of a Simple Non-cystoscopic Double-J Tube Removal Technique in Children

**DOI:** 10.3389/fped.2021.761903

**Published:** 2021-11-04

**Authors:** Qiao Bao, Weihua Lao, Tong Shi, Keyu Ouyang, Sai Ma, Wen Zhang, Yankun Lin

**Affiliations:** ^1^Department of Pediatric Urology, Guangdong Women and Children Hospital, Guangzhou, China; ^2^Department of Pediatric Surgery, Zhongnan Hospital of Wuhan University, Wuhan, China

**Keywords:** double-J ureteral stent, cystoscopy, non-cystoscopy, technique, feasibility

## Abstract

**Objective:** Double-J tube placement is an important procedure during upper urinary tract surgery. A primary drawback is the requirement of a second double-J tube removal under a cystoscope. Therefore, a simple and feasible alternative is required to remove the double-J tube without cystoscopy. The present study reported the feasibility and safety of a simple non-cystoscopic double-J tube removal technique.

**Method:** We retrospectively analysed children who underwent pyeloplasty and ureterovesical reimplantation between June 2015 and August 2021. A simple device (a catheter with a suture) was used to pull out the double-J tube. Patient characteristics, detailed surgical procedures, success and complication rates and reasons for failure were evaluated.

**Result:** A total of 613 children were included. The mean age of patients was 6.2 months (3 months−14 years). Non-endoscopic methods were used to remove the double-J tube in all except 6 patients (0.9%). Of the 6 patients who required ureteroscopy or cystoscopy, 4 had retraction of the double-J tube into the ureter, and 2 (0.6%) had bladder stones. Of the 613 patients, 479 (76.0%) required one attempt, 127 (20.1%) required two attempts and 19 (3.0%) required several attempts. No serious postoperative complications occurred in all patients. The most common complications were gross haematuria (22.5%), pain urinating (17.9%), difficulty in urinating (3.6%), foreskin injury (1.7%), and penile oedema (1.3%). No urethral strictures developed during the follow-up period.

**Conclusion:** The study results demonstrated that the modified and simple non-cystoscopic double-J tube removal technique is a safe and an effective alternative to cystoscopy in clinical practise.

## Introduction

Double-J tube removal is mostly performed under direct cystoscopic visualisation in the departments of urology and paediatric surgery. This method has a high success rate. However, the cystoscope requires sterile equipment and consumables. Factors such as damage to the cystoscope and cost of equipment maintenance should be considered for this procedure. Several new technologies and materials have been reported to facilitate the removal of the double-J tube through a non-cystoscopic approach. Some examples are as follows: the application of double-J tube with magnetic ends (1–4), surgical techniques for external placement of the double-J tube (5), and the percutaneous antegrade removal technique (6). However, these methods have certain limitations, such as the high cost of materials, requirement of technical expertise and additional surgical interventions, which increase the risk of complications. In this study, we reported a non-cystoscopic technique for double-J tube removal with a simple device of only a suture and one catheter. Our method has been reported in previous literature, such as the Vellore Catheter Snare technique (7) along with two similar techniques reported by Lin et al. (8) and Shao et al. (9). These studies have revealed that non-cystoscopic double-J tube removal had a shorter operation time and lower cost compared to that of a cystoscopic approach (7–9). However, these studies were performed with small sample size. Our study involved large sample size to assess the feasibility and safety of the non-cystoscopic double-J tube removal technique in children.

## Materials and Methods

### Study Population

This study was approved by the Guangdong Women and Children Hospital Ethics Committee (Guangdong, China). Written informed consent was obtained from all children or their parents after a complete explanation of the procedure. Patient records and information were anonymised before analysis. A total of 631 paediatric patients who presented with hydronephrosis and a history of pyeloplasty between June 2015 and August 2021 were admitted to our hospital. Ureterovesical reimplantation was performed, and double-J ureteral tubes were placed intraoperatively. All the children or their parents who agreed to undergo our modified double-J tube extraction without a cystoscope were included in our study.

### Surgical Techniques

**Figure 1:**
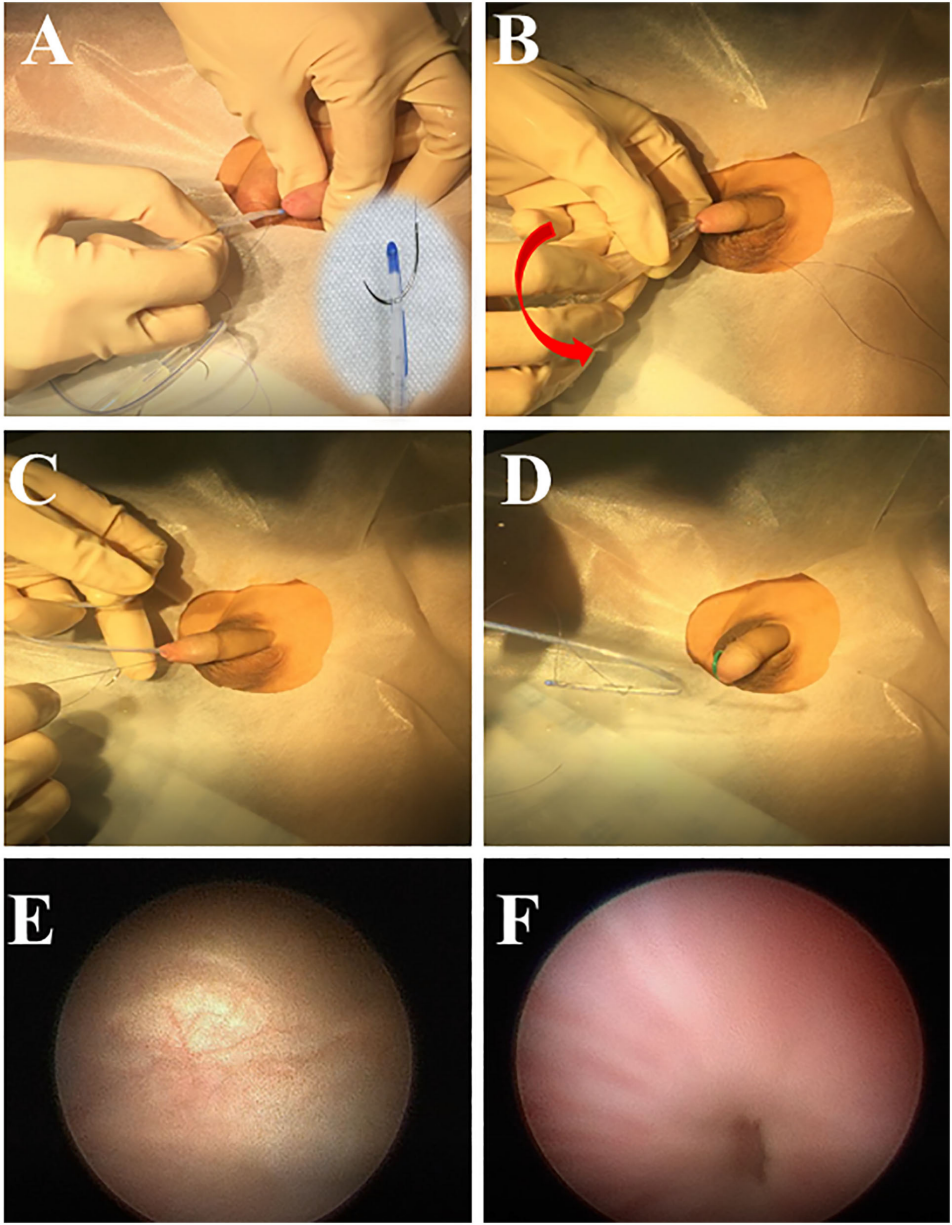
The surgical procedure of modified double-J ureteral tube removal. A 5–0 suture is threaded through the front opening of the Foley catheter, and the suture is folded back and placed into the bladder with the catheter **(A)**; the catheter is rotated **(B)**; the catheter is pulled out, and suture string is tensioned **(C)**; double-J tube is pulled out with the catheter and suture **(D)**; Re-examination of the bladder **(E)**; and urethral **(F)** injury after the non-cystoscopic removal of double-J tubes.

Most children included in this study were under 2 years of age. They did not have self-control; therefore, they had to be anaesthetised or sedated for double-J removal. The patients were placed in a supine position, and routine skin disinfection and draping were performed. The surgical procedure of our modified non-cystoscopic double-J tube removal technique (See the attached Supplementary Video file for detailed operation steps) was as follows: A string of 5–0 Monosyn^®^ suture was vertically threaded through the front opening of the F-6 (or F-8) Foley catheter. The Foley catheter was placed in the bladder along with the suture string after it was lubricated with proline or lidocaine cream (Figure 1A). The Foley catheter was inserted to the bladder and rotated several times (clockwise or counterclockwise; Figure 1B). The catheter was subsequently pulled out partially, and the suture string was tensioned (Figure 1C). When resistance was felt on the suture string, the suture and catheter were tightly pulled out of the urethra until the double-J tube was visible, and then it was removed gently (Figure 1D). If the tube was not visualised after pulling out the suture and Foley catheter, multiple attempts were made before switching to the cystoscopy removal. After the double-J tube was removed, an F-6 or F-8 Foley catheter was inserted and removed the next day. If the patients experienced difficulty in urinating after the removal of the catheter, they were recommended to keep the catheter indwelling for 2–3 days.

## Result

The modified non-cystoscopic double-J tube removal technique was performed in 631 patients between June 2015 and August 2021 (Table 1). The mean age was 6.2 months, ranging from 3 months to 14 years. A majority of the patients were boys (82.7%), and the right side was more involved (66.2%). A total of 537 patients presented with a history of pyeloplasty (83.5%).

**Table 1:** Patient demographics and related information of modified simple double J ureteral stent removal.

***N***	631
**Mean age (range)**	6.2 months (3 months−14 years)
**Sex**	
Boy	522 (82.7%)
Girl	109 (17.3%)
**Double J tube side**	
Right	418 (66.2%)
Left	210 (33.3%)
Both	3 (0.5%)
**Surgery history**	
Pyeloplasty	527 (83.5%)
Ureterovesical reimplantation	104 (16.5%)
**Successful pull out**	
One attempt	479 (76.0%)
Two tries	127 (20.1%)
Several attempts	19 (3.0%)
Unable to pull out need to switch to cystoscope	6 (0.9%)
**Cause of failure (Cases)**	
The double-J tube is retracted to the ureter	4 (0.6%)
Coexisting bladder calculi	2 (0.3%)
Complications^*^	
None	456 (72.3%)
Haematuria following catheter removal	142 (22.5%)
Pain urinating	113 (17.9%)
Difficulty urinating after pulling out the catheter	23 (3.6%)
The foreskin damage	11 (1.7%)
Hydrophallus	8 (1.3%)
**Preparation time (Minutes)**	2.55 ± 0.6
**Operating time (Seconds)**	61.81 ± 8.8
**Duration of hospital stay (Days)**	1.34 ± 0.3
**Time to regain automatic micturition (Hours)**	23.62 ± 1.5

* *Some children have multiple complications*.

In our study, the modified technique was unsuccessful in a few patients, who required cystoscopic removal (0.9%). Approximately 76% of the successful cases required one attempt, and 3% required two attempts. The technique was unsuccessful owing to the retraction of the double-J tube into the ureter and coexisting bladder calculi. Attempts made to pull out the double-J tube proved futile even after using a cystoscope. However, abdominal X-ray imaging revealed that the tube was observed to have retracted in the ureter instead of in the bladder. Eventually, a ureteroscope was used to pull the tube out. Simultaneously, bladder calculi caused resistance when the double-J tube was pulled out, leading to the failure of our technique.

The most common complication associated with this technique was gross haematuria, which accounted for approximately 22.5% of the cases. There was no case of severe bleeding. Gross haematuria was often accompanied by dysuria (17.9%). After the catheter was removed, a small number of patients experienced difficulty in urinating (3.6%), foreskin injury (1.7%) and penile oedema (1.3%).

The average pre-operation preparation and operation time with modified and simple double-J tube removal were 2.55 ± 0.6 min and 61.81 ± 8.8 s, respectively. The average duration of hospital-stay after the procedure was 1.34 ± 0.3 days. Three patients presented with gross haematuria after the withdrawal of Foley catheter with modified and simple double-J tube removal technique, compared to the 4 patients who underwent cystoscopy. There was no statistically significant difference (*P* > 0.05). The average time to regain micturition after the tube removal was 23.62 ± 1.5 h.

## Discussion

The double-J tube is one of the most widely used stents (10). The application of a double-J tube in disease conditions such as kidney stones, ureteral calculi, ureteropelvic junction obstruction, upper ureteral stricture, ureterovesical junction stenosis and ureteral distortion, injury has been increasing (10). A double-J tube provides an effective internal support and drainage. It can effectively relieve the upper urinary tract obstruction, protect renal function, replace renal fistulas and reduce postoperative infection and leakage (10–12). These developments have greatly expanded the scope of the application of double-J tubes in clinical practise. Despite several advantages of using a double J-tube, certain drawbacks still exist, an example of which is the need for a second operation to pull out the double-J tube (4, 13).

Double-J tube removal is mostly performed under direct cystoscopic visualisation. It has an almost 100% success rate. However, it requires specialised equipment, and the cost of the procedure is high. In clinical settings, re-sterilisation is required every time the equipment is used, preparation time for surgery is long, and the cost of maintenance is high. The present study used references from previous studies (7–9). In this procedure, a skilled clinician can have a high success rate and remove the tube in the first attempt. However, in this study, we failed to remove the tube in 6 patients (0.9%). Of these 6 patients, 4 underwent the procedure during the early period of the study, and failure was caused by the retraction of the tube into the ureter, which remained undetected owing to the absence of preoperative abdominal imaging examination. Therefore, routine abdominal X-ray imaging or ultrasonography should be performed before operation to avoid such failures.

Previous studies similar to this study have compared the conventional cystoscopic approach with the non-cystoscopic technique (8, 9). Lin et al. (8) reported 138 cases of the cystoscopic vs. non-cystoscopic technique with a mean operation time of 12.57 min vs. 5.05 min for male patients and 9.61 min vs. 4.63 min for female patients, respectively. Furthermore, Shao et al. (9) validated that the non-cystoscopic technique had a shorter operative time than that of the cystoscopic approach (7.40 ± 3.75 vs. 18.42 ± 2.77 min, respectively, *P* < 0.05). In addition, the mean cost for patients in the non-cystoscopic group was less than that for patients in the cystoscopic group ($736.70 ± 105.96 vs. $618.23 ± 110.31, respectively, *P* < 0.05). In this study, the mean operative time (including the preparation time) was ~5 min, which is shorter than that reported above. This may be associated with the fact that we currently have the maximum experience in performing this technique.

Currently, in our centre, the technique presented in this study is mainly used to pull out the double-J tube, and cystoscopy is rarely used. A drawback of this study is that the non-cystoscopic double-J tube removal was not simultaneously compared with cystoscopic double-J tube removal. Therefore, the purpose of this study was not to speculate the difference between the two surgical methods in terms of operation time and treatment costs. However, this non-cystoscopic technique only requires a catheter with sutures. The material is simple and cheap, and the preparation is easy, which undoubtedly shortens the operation time and reduces the cost. Currently, the total cost of taking out the double-J tube under a cystoscope at our centre is CNY 1187 (~$182.7). However, our technology only costs CNY 200 ($30.7). This cost excludes the total cost of the patient's hospital stay.

In this study, the incidence of mild complications was estimated to be 27%. The most common complication was gross haematuria, which lasted for a short duration and disappeared after urinating one or twice. It was observed that the occurrence of gross haematuria was associated with the number of operative attempts. The higher the number of failed attempts, the higher the incidence of gross haematuria. Therefore, to avoid urethral and bladder injury, multiple attempts were avoided. In this study, the double-J tube was pulled out successfully after two attempts in 96% of the patients. The possibility of a patient incurring serious urethral or bladder injury, leading to long- term urethral stricture, had been a concern. Therefore, we performed cystoscopy after the non-cystoscopic technique for some children and observed that there was no serious injury (Figures 1E,F), and no patient had a urethral stricture in the long-term follow-up.

The requirement of anaesthesia is another drawback. Most patients included in our study were children under 1 year of age. Therefore, if anaesthesia or sedation was not administered before operation, it would have been difficult to complete the procedure. Whether it is traditional cystoscopy or other transurethral operations, without anaesthesia, it is certain to cause discomfort in children, inconvenience in operation and stress in the family members of patients. Initially, we attempted performing the procedure without anaesthesia in older children; however, we found it impossible. Therefore, currently, we anaesthetise or sedate the patients before pulling out the double- J tube at our centre.

Previously, between 2015 and 2016, patients were usually kept in the hospital for an extra day after the procedure for observation because a catheter was usually inserted after the removal of the double-J tube, thus increasing the chance of contracting a urinary tract infection. Recently, owing to the experience attained in performing this procedure over the years, patients are discharged after the procedure. Moreover, the practise of indwelling a catheter after the procedure has been abolished.

To improve the success rate of double-J tube removal with the approach presented in this study, some key points are as follows: (1) Both Foley catheter and suture string require lubrication with sterile proline to avoid abrasion of the mucous membrane, which further prevents post-operational haematuria and dysuria. Lidocaine can be used to lubricate and anaesthetise the urethral mucous membrane, if necessary. (2) The operation irritates the mucous membrane of the bladder and urethra. General anaesthetics or sedation are recommended to eliminate the fear and non-cooperation of paediatric patients. If general anaesthetics are contraindicated, oral sedatives and surface anaesthetics can be used. (3) A moderate amount of urine should be left in the bladder before removing the tube to provide sufficient space for the removal of the catheter. (4) If the tube is placed on the left or right, the Foley catheter should be rotated counterclockwise or clockwise, respectively. The suture string should be tensioned before slowly removing the Foley catheter when resistance is felt. (5) During the operation, if the F-6 Foley catheter is enwound by 5–0 suture or if the tension is not enough, the F-8 Foley catheter can be used to continue the procedure. (6) Initially, we did not place a Foley catheter after removing the tube, which resulted in patients presenting with dysuria. To avoid post-operational dysuria, an F-6 or F-8 Foley catheter was routinely placed and removed the next day. (7) When multiple attempts are required, cystoscopy should be used to avoid excessive damage to the urethral and bladder mucous membrane owing to the rotation of the Foley catheter. In the earlier period, three attempts were the cut-off value. If the double-J tube could not be pulled out after three attempts using our method, a cystoscope was used to aid the removal. At most, nine attempts were made to pull out the double-J tube; therefore, the number of attempts required in some cases depends on the clinical experience.

The limitation of this study is that there is no multi-centre verification of our modified tube removal technique. However, this method is routinely performed at our centre and has been implemented by several paediatric urologists with different work experiences. In addition, previous studies with small sample size have also reported the approach as feasible and safe (7–9). However, this study included large sample size; therefore, it is clinically significant and effective for application and promotion.

## Conclusion

In this study, we presented a modified non-cystoscopic double-J ureteral tube removal technique in children, which was effective and safe. The data from our single-centre and large cohort study supported the practicability of this simple procedure. This technique is being used in multiple centres; however, feedback from other centres is required to further validate our findings.

## Data Availability Statement

The original contributions presented in the study are included in the article/Supplementary Material, further inquiries can be directed to the corresponding author/s.

## Author Contributions

QB and WL: manuscript writing and data analysis. TS, KO, and SM: data collection. TS, YL, and WZ: study supervisors and manuscript revision. All authors contributed to the article and approved the submitted version.

## Conflict of Interest

The authors declare that the research was conducted in the absence of any commercial or financial relationships that could be construed as a potential conflict of interest.

## Publisher's Note

All claims expressed in this article are solely those of the authors and do not necessarily represent those of their affiliated organizations, or those of the publisher, the editors and the reviewers. Any product that may be evaluated in this article, or claim that may be made by its manufacturer, is not guaranteed or endorsed by the publisher.
